# Unique Role of Histone Methyltransferase PRDM8 in the Tumorigenesis of Virus-Negative Merkel Cell Carcinoma

**DOI:** 10.3390/cancers12041057

**Published:** 2020-04-24

**Authors:** Elias Orouji, Wiebke K. Peitsch, Azadeh Orouji, Roland Houben, Jochen Utikal

**Affiliations:** 1 Skin Cancer Unit, German Cancer Research Center (DKFZ), 69120 Heidelberg, Germany; 2 Department of Dermatology, Venereology and Allergology, University Medical Center Mannheim, Ruprecht-Karl University of Heidelberg, 68167 Mannheim, Germany; 3 Department of Genomic Medicine, University of Texas MD Anderson Cancer Center, Houston, TX 77054, USA; 4 Department of Dermatology and Phlebology, Vivantes Klinikum im Friedrichshain, 10249 Berlin, Germany; 5 Department of Dermatology, Venereology and Allergology, University Hospital Würzburg, 97080 Würzburg, Germany

**Keywords:** histone, histone methyltransferase PRDM8, chromatin regulator, EGR1, miRNA, MCV-negative, Merkel cell carcinoma, MCPyV

## Abstract

Merkel cell carcinoma (MCC) is a deadly skin cancer, and about 80% of its cases have been shown to harbor integrated Merkel polyomavirus in the tumor cell genome. Viral oncoproteins expressed in the tumor cells are considered as the oncogenic factors of these virus-positive Merkel cell carcinoma (VP-MCC). In contrast, the molecular pathogenesis of virus-negative MCC (VN-MCC) is less well understood. Using gene expression analysis of MCC cell lines, we found histone methyltransferase PRDM8 to be elevated in VN-MCC. This finding was confirmed by immunohistochemical analysis of MCC tumors, revealing that increased PRDM8 expression in VN-MCC is also associated with increased H3K9 methylation. CRISPR-mediated silencing of PRDM8 in MCC cells further supported the histone methylating role of this protein in VN-MCC. We also identified miR-20a-5p as a negative regulator of PRDM8. Taken together, our findings provide insights into the role of PRDM8 as a histone methyltransferase in VN-MCC tumorigenesis.

## 1. Introduction

Merkel cell carcinoma (MCC) is a lethal skin cancer, frequently associated with the Merkel cell polyomavirus (MCV), which is found clonally integrated in about 80% of cases [1,2]. However, there is little understanding about the underlying tumorigenesis mechanisms of virus-negative MCC (VN-MCC). It has been recently reported that these tumors, when compared to virus-positive MCC (VP-MCC) usually harbor a significant mutational burden and are more prevalent in areas with higher sun exposure [3,4,5]. Epidemiological data support clinically relevant differences between the two subtypes [6], suggesting even different cells of origin for them. 

Integration of MCV genome into the host cells and accidental fragmentation of the genome during this process, leading to the oncogenic transformation by MCV, is the proposed mechanism of tumorigenesis in VP-MCC. Although no particular host for the virus has been identified in VP-MCCs, there are studies suggesting blood monocytes as a route of dissemination of MCV in the human body or for keratinocytes to be the skin cells that are infected by the virus [7,8]. However, to date there is no conclusive evidence on cells of origin in MCC cells [9,10,11]. The other MCC subtype are virus-negative tumors. Higher copy number variations (CNV), mutational burden representing UV signature, and inactivation of tumor suppressor genes through (epi)genetic mechanisms are more frequently seen in VN-MCC [5,12].

Histone methylation is known to regulate chromatin structure through various mechanisms including transcriptional activation or repression. Several histone-modifying enzymes are responsible for (de)methylation in cells, leading to tumor initiation or progression [13].

In this study, we explored the transcriptional landscape in MCC subtypes with the focus on histone-modifying enzymes. We identified the PRDM8 gene encoding a SET-domain containing protein from the family of histone methyltransferases to be highly expressed in VN-MCC compared to the virus-positive subtype [14]. We further investigated the mechanism by which PRDM8 is regulated in this type of tumor that differentiates VN-MCC from VP-MCC tumors, and lastly the mechanism by which it acts in VN-MCC. 

## 2. Methods

### 2.1. Patient Samples and MCC Cell Lines

A panel of cell lines derived from patients with histologically confirmed MCC diagnosis was used. VP-MCV cell lines included PeTa, WaGa, MKL1, MKL2, and LoKe [15,16,17] and VN-MCV cell lines were MCC13, MCC26, and UISO [18,19,20,21]. MCC cell lines were cultured in RPMI-1640 supplemented with 10% fetal bovine serum, 100 U/mL penicillin, and 0.1 mg/mL streptomycin. 

Formalin-fixed paraffin-embedded (FFPE) tumor tissue specimens from a cohort of MCC patients were investigated in this study. Samples were obtained from patients referred to the Department of Dermatology, Venereology and Allergology, University Medical Center Mannheim, Heidelberg University during 2001–2015 (ethics committee vote: 2014-835R-MA).

### 2.2. Immunohistochemistry (IHC), Immunocytochemistry, and Western Blot

FFPE blocks of MCC tumors were cut into 5µm sections. Sections were pretreated and followed by antigen epitope retrieval using citrate buffer. IHC was performed manually using standard protocol as described previously [22]. Anti-PRDM8 antibody (mouse monoclonal IgG2b PRDM8 antibody; Santa Cruz, sc-390001), anti-H3K9me1 (rabbit polyclonal to histone H3, mono methyl K9; Abcam, ab8896), anti-H3K9me2 (mouse monoclonal to histone H3, di methyl K9; Abcam, ab1220), and anti-H3K9me3 (rabbit polyclonal to histone H3, tri methyl K9; Abcam, ab8898) were used for immunostaining (IHC and ICC) as well as Western blotting. Immunostaining sections were scored using our previously described method, which incorporates both quantity as well as the intensity of the staining [22]. Analysis of immunostaining sections was performed by two independent investigators (E.O. and J.U.).

To perform Western blot, cells were lysed in RIPA lysis buffer (ThermoFisher, Waltham, MA, USA), and centrifuged at 15,000× *g* for 20 min at 4 °C. Protein was assayed using a Pierce BCA Protein Assay Kit according to the manufacturer’s protocol. A total of 30–100 μg of protein was run on gel. Membranes were blocked for 1 hour at room temperature (RT) with Odyssey blocking buffer (LI-COR, Lincoln, NE, USA). Membranes were then incubated with the primary antibodies (anti-PRDM8, sc-390001; anti-H3K9me3, ab8898; anti-histone H3, ab1791) overnight at 4 °C, followed by 1 hour incubation at RT with IRDye 800 secondary antibodies (LI-COR). Membranes were washed three times in phosphate-buffered saline (PBS) containing 0.01% Tween-20 for 5 min between each step. Blots were scanned, and proteins were detected using Odyssey Imaging System (LI-COR).

### 2.3. mRNA and miRNA Expression Analyses 

Total RNA or small-sized RNA was isolated from cell lines using RNeasy Mini Kit or miRNeasy Mini Kit (Qiagen, Germantown, MD, USA) respectively, per the manufacturer’s protocol. Size and quality of RNA samples were measured using Agilent 2100 Bioanalyzer. Gene expression profiling was carried out using Illumina whole genome BeadChip Sentrix array, HumanHT-12 v4 platform. miRNA profiling was performed using SurePrint human miRNA microarrays. Data were normalized and analyzed using Chipster 2.9.X. False discovery rate (*FDR*) < 0.05 was used as statistical significance throughout the analysis. 

### 2.4. CRISPR-Cas9-Mediated Gene Silencing

To target PRDM8, we designed single guide RNAs (sgRNAs) using GPP web portal from the Broad Institute (https://portals.broadinstitute.org/gpp/public/analysis-tools/sgrna-design) (sgPRDM8: ATAAGTCCCCAAGACGAACA, PAM: AGG). Oligos were annealed and ligated into the lentiCRISPR v2 construct (Addgene; plasmid #52961). Products were confirmed by Sanger sequencing. Cells were infected with this vector and then were selected by puromycin for 7 days. To assess performance of clustered regularly interspaced short palindromic repeats (CRISPR) in PRDM8 disruption, PCR using primers (Fwd: 5′ GCCATCCACAGACTTCCACA 3′, Rev: 5′ GCCTTCTTCACCTCCACGAA 3′) to target PRDM8 was performed.

### 2.5. Chromatin Immunoprecipitation Sequencing (ChIP-seq) and ChIP-qPCR

Approximately 2 × 10^7^ cells from MCC cell lines (MCC13^CTRL^, MCC13^PRDM8-KD^) were crosslinked for 10 min using PBS/1.1% formaldehyde. The crosslinking reaction was blocked with 10 min incubation in 1/20 volume of 2.5 M glycine. Cells were then washed three times with ice-cold PBS, collected, and centrifuged at 1000× *g* at 4 °C. Cell pellets were resuspended in 1 ml lysis buffer 1 (LB1: 50 mM HEPES-KOH, pH 7.5; 140 mM NaCl; 1 mM Ethylenediaminetetraacetic acid (EDTA); 10% glycerol; 0.5% NP-40; 0.25% Triton X-100; supplemented with protease inhibitors) and incubated for 10 min at 4 °C. After the incubation, the nuclei were centrifuged at 1000× *g* at 4 °C, and the supernatant was discarded. Cells were washed in 1 mL cold lysis buffer 2 (LB2: 10 mM HEPES-KOH, pH 8.0; 200 mM NaCl; 1 mM EDTA; 0.5 mM EGTA). Nuclei were washed twice in cold lysis buffer 3 (LB3: 10 mM HEPES-KOH, pH 8.0; 200 mM NaCl; 1 mM EDTA; 0.5 mM EGTA; 0.1% sodium deoxycholate; 0.5% sodium lauroyl sarcosine) and resuspended in 500–1000μl LB3. Then, chromatin was sonicated at 4 °C using a Covaris S220 focused ultrasonicator at the following settings: 30 min shearing, duty cycle 20%, intensity 5 for 200 cycles per burst. After shearing, cellular debris was removed by centrifugation at 16,000× *g* for 5 min. Shearing efficiency was tested by DNA concentration assessment and fragment-size distribution analysis on small aliquots of the supernatants. Sonicated chromatin was then immunoprecipitated with DiaMag protein A-coated magnetic beads (Diagenode, Denville, NJ, USA) using PRDM8 antibody (sc-390001). Immunoprecipitated DNA was then isolated by digestion with proteinase K at 65 °C for 4 h and subsequently purified using AMPure XP beads. Chromatin immunoprecipitation (ChIP) libraries were constructed using NEBNext Ultra II DNA Library Prep Kit for Illumina according to standard protocol [23]. ChIP libraries were sequenced using Illumina HiSeq2500. Generated Fastq files were aligned to reference human genome (hg19) using Burrows-Wheeler Aligner (BWA), and MACS2 was used for peak calling. Bigwig files were generated for the visualization using Integrative Gene Viewer (IGV) [23].

For ChIP-qPCR, specific primers were designed to target the binding sites around the transcription start site (TSS) of the early growth response protein 1 (EGR1), YY1, and CHAMP1 in the PRDM8 chromatin immunoprecipitated and input samples. Potential binding site sequences were split into ≈400 bp bins (S_1_-S_N_). Primer sequences are provided in the Supplementary Materials section. 

### 2.6. ChIP-seq Visualization Using IGV Browser

Input-subtracted, whole-genome coverage ChIP-seq tracks of PRDM8 as well as H3K4me3 from GEO (GSM1711864) were used for visualization on IGV. 

### 2.7. Quantitative PCR

Total RNA was isolated using the RNeasy Mini Kit (Qiagen). Synthesis of complementary DNA (cDNA) was performed using the SuperScript III First-Strand Synthesis System (Life Technologies, Carlsbad, CA, USA). PCR quantification was performed using the SYBR green method. The following primer sequences were used to detect human PRDM8 transcripts: PRDM8_fwd: TTACACCACCTGCGACATCC; PRDM8_rev: TGCTGAGGTGTCTACCCGAA. GAPDH was used as the control, using the following primers: GAPDH_fwd: CCTGCACCACCAACTGCTTA; GAPDH_rev: GGCCATCCACAGTCTTCTGAG. The efficiency of qPCR primers for each primer pair was determined by serial dilutions of cDNA. Values were normalized to GAPDH using the ΔΔCt analysis method.

### 2.8. Colony Formation Assay

About 2000 cells were plated in each well of a 24-well culture plate and were incubated for 3–5 days at 37 °C. Media was removed and colonies were fixed and stained with 0.6% w/v methylene blue in methanol. The experiment was repeated three times.

### 2.9. miRNA Overexpression

hsa-miR-20a-5p mimic and negative control miRNA were purchased (Active Motif; MIM0050—UAAAGUGCUUAUAGUGCAGGUAG). The mimic has the same sequence of endogenous miR-20a-5p. Negative control miRNA is a non-specific oligonucleotide that has a random sequence. Cells were transfected with 25 nM miRNAs in 5 μL lipofectamine 2000 (ThermoFisher) and were grown for 24 h without antibiotics. Then, media was replaced by fresh media supplemented with 100 U/mL penicillin and 100 ng/mL streptomycin.

### 2.10. Analysis of ChIP-seq Data from ENCODE Database

We used Search Candidate cis-Regulatory Elements by ENCODE (SCREEN) portal from the Encyclopedia of DNA Elements (ENCODE), which is an integrative level of data generated from multiple ground-level annotations. The core of this level is the Registry of candidates cis-Regulatory Elements (ccREs) and SCREEN. Using this web portal, we filtered skin tissue samples and provided PRDM8 genomic coordinates to obtain transcription factors (TFs) that bind to this region on the basis of the ChIP-seq data stored in ENCODE database. Next, we overlapped these TFs using UpSetR, an R package for the visualization of intersecting sets.

## 3. Results

### 3.1. PRDM8 Is Highly Expressed in Virus-Negative Merkel Cell Carcinoma Cells and Is Correlated with Tumor Proliferation and Clonogenicity 

**Figure 1:**
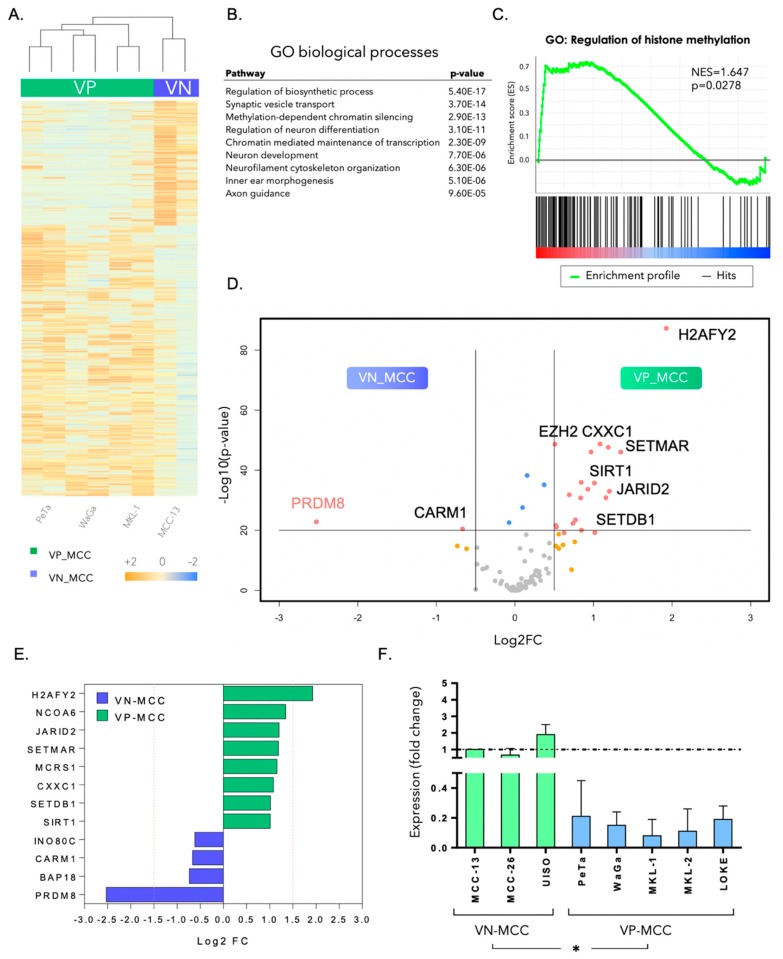
(**A**) Heatmap of the differentially expressed genes (DEGs) in virus-negative (VN) versus virus-positive (VP) Merkel cell carcinoma (MCC) cell lines. Color scale bar shows expression fold change. (**B**) Pathway analysis shows deregulated pathways in two subtypes of MCC. Methylation-dependent chromatin silencing and chromatin-mediated maintenance of transcription are among the top enriched Gene Ontology (GO) biological terms (*p*-value 2.9 × 10^−13^ and 2.3 × 10^−9^, respectively). (**C**) Gene set enrichment analysis (GSEA) shows differential enrichment of regulation of histone methylation in two subtypes of MCC (NES = 1.647, *p* < 0.05). (**D**) Volcano plot shows expression level of histone-regulating genes in VN-MCC compared to VP-MCC. *x*-axis shows the Log2 fold change of gene expression in VP vs. VN and *y*-axis shows the −Log10 of the *p*-value. The upper left corner shows the top upregulated histone-modifier genes in VN-MCC, whereas the upper right corner shows upregulated histone-modifier genes in VP-MCC. (**E**) Bar plot shows the Log2 fold change of the top upregulated genes in VN-MCC (blue bars) and the top upregulated genes in VP-MCC (green bars). (**F**) Expression data for PRDM8 (the top upregulated gene in VN-MCC) assessed using quantitative PCR in three VN-MCC cell lines and five VP-MCC cell lines. * *p*-value < 0.05.

Gene expression profiling was performed in four Merkel cell carcinoma cell lines, using the Illumina RNA microarray platform. Differentially expressed genes (DEGs) were identified in VN versus VP MCC. Figure 1A shows the top 1000 differentially expressed genes in MCC subtypes (*FDR* < 0.05). To investigate enriched pathways in each of the MCC subtypes, we performed pathway analysis using the gene expression dataset. Figure 1B demonstrates the Gene Ontology (GO) biological processes involved in differentially expressed genes in VN-MCC vs. VP-MCC. Among the top deregulated GO terms, there were methylation-dependent chromatin silencing as well as chromatin-mediated maintenance of transcription. We further performed gene set enrichment analysis (GSEA) in the MCC expression dataset, and enrichment for histone methylation regulation was observed (Normalized enrichment score (NES) = 1.647, *p* < 0.05) (Figure 1C). Therefore, we focused on the chromatin regulation and histone methylation complexes in either of the MCC subtypes. To this end, we curated a shortlist of 107 chromatin and histone regulatory genes by combining two gene sets from the Molecular Signatures Database (MSegDB, v6.2): histone methyltransferase complexes (GO: 0035097) and genes with histone methyltransferase activity (GO: 0042054). The top differentially expressed genes from this list were identified (*FDR* < 0.05). Several genes were found to be increased in VP-MCC (Figure 1D upper right quadrant of the plot), whereas only a few genes demonstrated higher expression levels in VN-MCC (Figure 1D upper left quadrant). Figure 1E shows a shortlist of differentially expressed genes in each of the MCC subtypes, with PRDM8 standing out as being most significantly increased in VN-MCC. To validate this finding, we performed qPCR to detect PRDM8 transcripts in eight MCC cell lines from both VP and VN subgroups. A significantly higher expression of PRDM8 was detected in VN-MCC cell lines (*p* < 0.05; Figure 1F and Figure S1A). 

**Figure 2:**
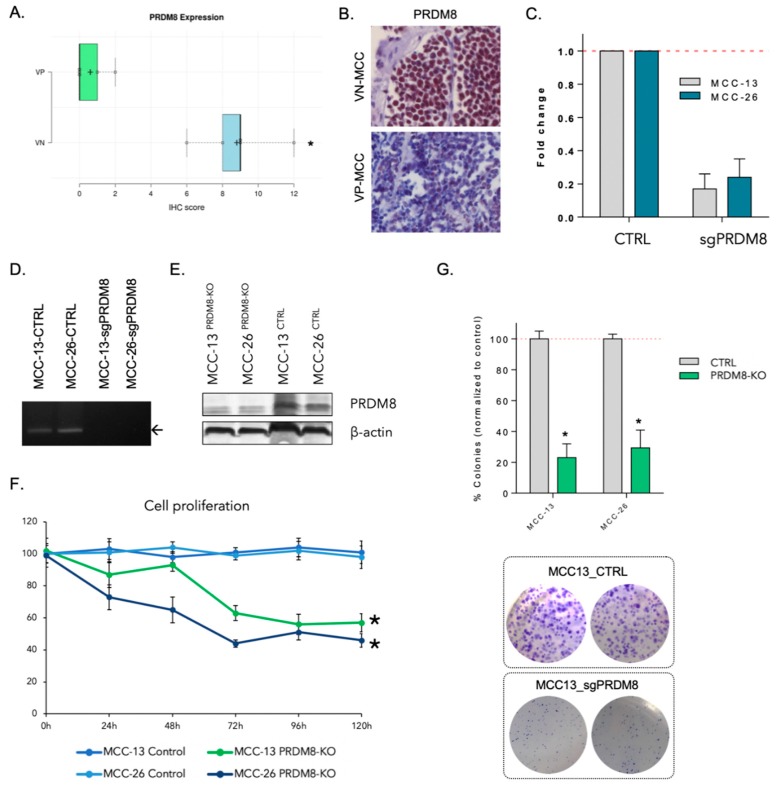
(**A**) Analysis of immunohistochemistry (IHC) performed in VN- and VP-MCC patient tumors, indicating higher overall score in VN-MCC compared to VP-MCC (*p* < 0.05). (**B**) Representative PRDM8 IHC in a VN-MCC vs. a VP-MCC tumor (20× magnification). (**C**) Knocking down PRDM8 using CRISPR-Cas9 in two VN-MCC cell lines resulted in lower expression levels of this gene in both cell lines. (**D**) Image of the agarose gel from the PCR experiment targeting PRDM8, confirming depletion of this gene in the cell lines. (**E**) Western blot performed on PRDM8-KO and control VN-MCC cell lines. β-actin was used as an internal positive control. (**F**) Cell proliferation experiment performed in two VN-MCC cell lines, indicating a decrease in proliferation rate upon PRDM8 knockdown. Asterisks show *p*-value < 0.05. (**G**) Clonogenicity assay performed in VN-MCC cells, indicating a lower number of colonies in the PRDM8-depleted cells. Asterisks show *p*-value < 0.05. Representative figures of MCC13 cells are shown.

To investigate whether patient tumor samples recapitulated a similar pattern as in MCC cells, we analyzed five tumors from each MCC subtype for their PRDM8 expression. Immunohistochemistry revealed a significant increase in PRDM8 expression in VN- versus VP-MCC (IHC overall score 8.8 ± 2.16 vs. 0.6 ± 0.89, *p* < 0.05). IHC sections were analyzed for both intensity and quantity of positive cells. (Figure 2A and Figure S1C). Figure 2B shows PRDM8 expression in representative patient tumor samples from VP and VN subtypes. 

To explore the functional role of PRDM8 in VN-MCC, we performed CRISPR-mediated gene silencing of PRDM8 in MCC13 and MCC26 cell lines (two VN-MCC cell lines) and validated CRISPR efficiency at gene and RNA expression level (Figure 2C,D). Expression of PRDM8 protein was also tested using Western blot on these VN-MCC cell lines, indicating significantly lower expression in PRDM8-KO (knock-out) cells (Figure 2E).

Proliferation assay was performed in the PRDM8 knockout cells and was compared to the parental cell lines. Both MCC13 and MCC26 knockout cell lines demonstrated lower proliferation rates compared to the intact cells (Figure 2E). Indeed, clonogenicity assessment of PRDM8-depleted cells to the control cells indicated a major decrease in the clonogenic capacity of PRDM8-edited cells (Figure 2F).

### 3.2. PRDM8 Expression Was Correlated with Global H3K9me3 Level in VN-MCC

**Figure 3:**
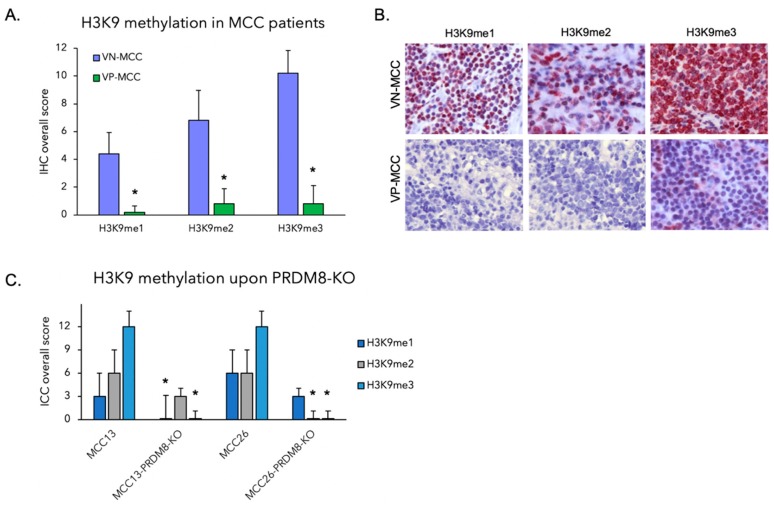
(**A**) H3K9 immunohistochemistry performed in a stepwise manner (H3K9me1, H3K9me2, and H3K9me3) comparing global expression of these repressive histone marks in VN-MCC and VP-MCC tumors. Significantly higher expression levels of methylated H3K9 were observed in VN-MCC tumors. Asterisks show *p*-value < 0.05. (**B**) Representative methylated H3K9 (me1, me2, and me3) IHC in a VN-MCC and a VP-MCC tumor (20× magnification). (**C**) Immunocytochemistry (ICC) performed on MCC-13 and MCC-26 cell lines and their corresponding PRDM8-KO cells, indicating significantly lower levels of H3K9me3 upon PRDM8 depletion (* *p* < 0.05).

PRDM8 is reported as a methyltransferase preferentially acting on Lys-9 of histone H3 (H3K9) [14]. Histone H3K9 methylation is widely considered as a mark of transcriptional repression. Therefore, we analyzed PRDM8 expression along with the H3K9 methylation (mono-, di-, and trimethylation) in five VN-MCC versus five VP-MCC tumors using immunostaining. Immunohistochemistry revealed significantly higher levels of methylated H3K9 in VN-MCC cells compared to VP-MCC. Highest differential levels were observed in trimethylated H3K9 (Figure 3A,B). To investigate whether the correlation of PRDM8 expression level with H3K9 methylation level was functionally relevant, we utilized PRDM8-depleted VN-MCC cell lines (MCC-13 and MCC-26) and the control cells and tested them against H3K9me3 antibody. Interestingly, we noticed that CRISPR-mediated silencing of PRDM8 decreased global level of H3K9me3 in these cells (Figure 3C and Figure S1B). This can shed light on the PRDM8 mechanism of action by possibly silencing tumor suppressors through the recruitment of repressive histone mark H3K9me3. As shown in Figure 2F,G, this will lead to higher tumor proliferation and increase in tumor clonogenic capacity.

### 3.3. miR-20a-5p Regulated PRDM8 in VN-MCC

Next, we were interested in investigating upstream regulators of PRDM8 in Merkel cell carcinoma. 

We sought mechanisms that can impact lower expression level of PRDM8 in VP-MCC subtype. Therefore, due to the known role of miRNAs in virus-mediated oncogenesis, we set out to investigate miRNA regulation by profiling and analyzing miRNA expression in VN-MCC versus VP-MCC cells as another mechanism that can govern PRDM8 RNA expression in MCC.

**Figure 4:**
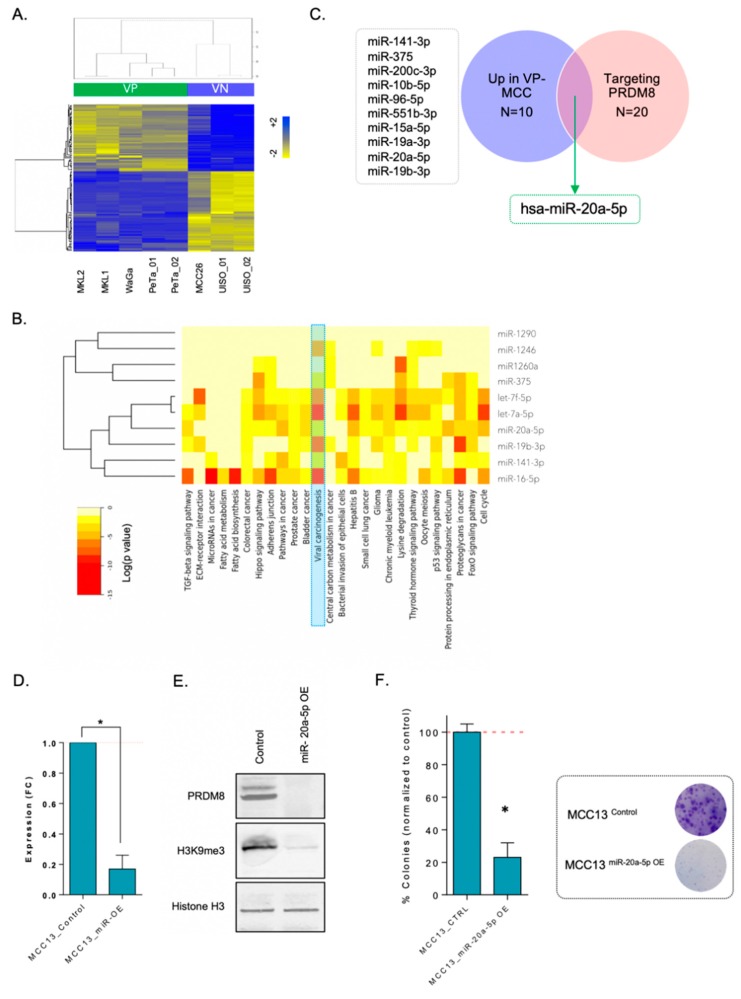
(**A**) Heatmap shows differentially expressed microRNA (miRNAs) in VP- vs. VN-MCC cell lines from microarray data analysis (false discovery rate (*FDR*) <0.05). (**B**) miRNA pathway analysis using mirPath v.3 in the top differentially expressed miRNAs, showing enriched pathways (i.e., viral carcinogenesis and miRNAs involved in cancer). (**C**) Intersection of top upregulated miRNAs in VP-MCC with top miRNAs that could potentially target PRDM8 (scored using miRWalk) indicates miR-20a-5p as the only miRNA that can target PRDM8 and at the same time is downregulated in VN-MCCs. (**D**) Overexpressing miR-20a-5p using miRNA mimic decreases PRDM8 expression level significantly in MCC13 cells. (**E**) Immunoblot shows that PRDM8 protein levels are decreased upon miR-20a-5p overexpression. Ectopic overexpression of miR-20a-5p also led to the decrease in H3K9me3 levels. Histone H3 is used as the control. (**F**) Colony formation assay performed in MCC13 cells compared to miR-20a-5p-overexpressed MCC13 cells indicated lower clonogenicity capability upon overexpression of miR-20a-5p. Representative figures of colonies in MCC13 cells are shown. * *p*-value < 0.05.

Figure 4A demonstrates differentially expressed miRNAs in virus negative vs. positive MCC cells in five VP-MCC and three VN-MCC samples. Next, we performed pathway analysis using miRPath v3.0 on the differential miRNAs in MCC subtypes. Figure 4B shows the signaling pathways regulated by the top deregulated miRNAs in VN- vs. VP-MCC cells. Interestingly, pathways such as “viral carcinogenesis” were enriched, as highlighted in the heatmap (Figure 4B) [24]. We then identified the top 10 miRNAs that were upregulated in VP-MCC and downregulated in VN-MCC samples. Next, we overlapped top deregulated miRNAs with a list of miRNAs that can target PRDM8 (ranked using miRWalk) [25]. This analysis helped us to identify PRDM8-targeting miRNAs that were lost in VN-MCC, and as a result, PRDM8 levels were increased. As shown in Figure 4C, hsa-miR-20a-5p was the only miRNA that appeared in the intersection. We therefore hypothesized that miR-20a-5p could target PRDM8 in VP-MCC and decrease the PRDM8 expression and suppress the function of this protein. To test this hypothesis, we overexpressed miR-20a-5p in VN-MCC cell line (MCC13) using miRNA mimics of miR-20a-5p and explored its impact on PRDM8 expression. We found out that overexpressing miR-20a-5p in MCC13 cells reduced PRDM8 expression significantly (*p* = 0.032), supporting the notion of PRDM8 being regulated by miR-20a-5p (Figure 4D). We next examined whether downregulating PRDM8 through ectopic overexpression of miR-20a-5p might have impacts on H3K9 methylation. Interestingly, miR-20a-5p-overexpressing MCC13 cells had a significantly lower H3K9me3 expression level (Figure 4E), which is in line with our prior finding of a global decrease of H3K9me3 levels upon CRISPR-mediated PRDM8 depletion in VN-MCC cells. Lastly, we tested the clonogenic capacity of MCC13 control cells and their corresponding miR-20a-5p-overexpressing cells and observed reduction of colony formation capability in miR-20a-5p-overexpressing cells, which confirmed the crucial role of PRDM8 in the tumorigenicity of VN-MCC cells (Figure 4F).

### 3.4. PRDM8 Acted through Its Partnership with EGR1 

To further explore PRDM8 mechanism of action, we investigated transcription factors (TFs) that could potentially interact with the PRDM8 binding sites throughout the genome. Due to the involvement of TFs in activation of different signaling pathways, identification of TFs that can bind to PRDM8 can help to pin down activated pathways, hence elucidating the role of PRDM8 in VN-MCC. To this end, we employed available ChIP-seq data from the ENCODE database. ChIP-seq datasets from both fibroblasts and keratinocytes were included in the analysis. These are the two possible skin cell types that could be considered as possible cells of origin in MCC. To generate a list of TFs with high binding affinity to PRDM8, we used Search Candidate cis-Regulatory Elements by ENCODE (SCREEN) portal from the Encyclopedia of DNA Elements (ENCODE), which is an integrative level of data generated from multiple ground-level annotations. Next, by providing PRDM8 genomic coordinates and selecting available skin samples, we obtained TFs that bind to this region. This analysis was performed by genome-wide interrogation of available TF ChIP-seq data stored in the ENCODE database. Next, we overlapped these TFs using UpSetR, an R package for the visualization of intersecting sets.

**Figure 5:**
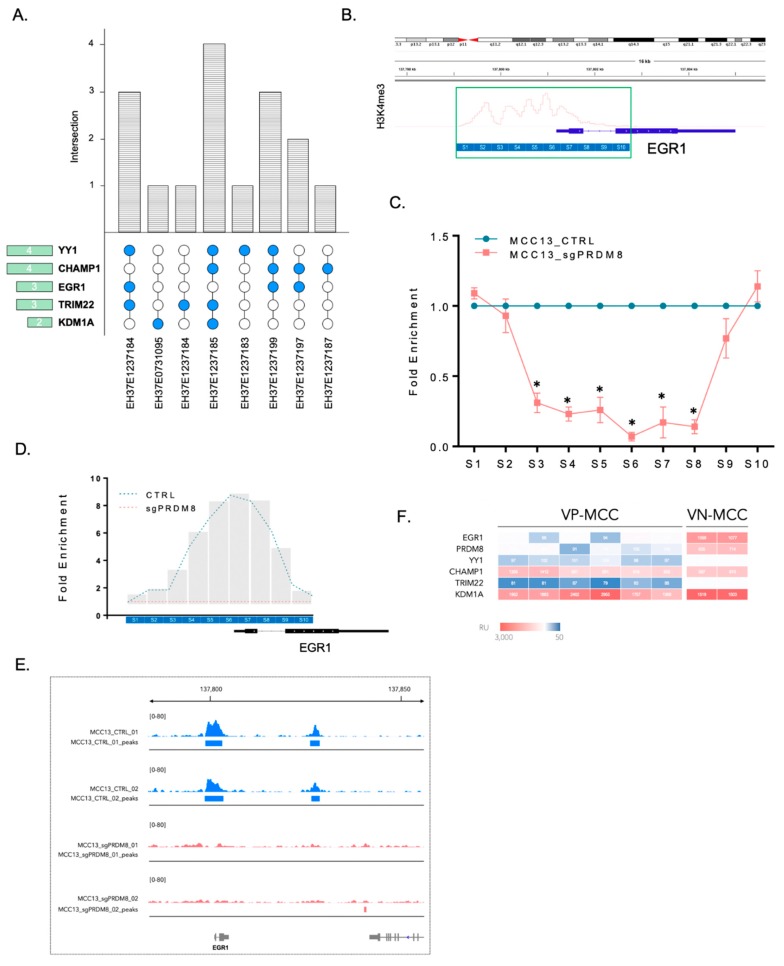
(**A**) UpSetR plot showing intersection of transcription factors (TFs) with high binding affinity to PRDM8 from the chromatin immunoprecipitation sequencing (ChIP-seq) data of Encyclopedia of DNA Elements (ENCODE) database. Sample IDs including both fibroblasts and keratinocytes as cells of origin in MCC are indicated in lower part of the plot. Blue circles show samples with high binding affinity of TF-PRDM8 and white circles are samples with no TF-PRDM8 binding. Gray bars show the number of top-scored TFs that bind to PRDM8. Aggregate number of samples for each row is shown in the green horizontal bar next to each TF. (**B**) Integrative Gene Viewer (IGV) browser snippet of H3K4me3 ChIP-seq track of the MCC cells, showing promoter binding site around the transcriptional start site (TSS) of early growth response protein 1 (EGR1). Chromosome ideogram and coordinates are shown in the upper panel. Binding site DNA sequence was split into ≈400bp sites (S_1_-S_10_) for further enrichment analysis using primers specific to each site. (**C**) PRDM8-depleted and control cells (MCC13_sgPRDM8 and MCC13_CTRL) underwent PRDM8 chromatin immunoprecipitation followed by qPCR (ChIP-qPCR) for enrichment at each binding site. PRDM8-depleted cells showed significantly lower binding enrichment in bins S_3_ to S_8_ compared to control cells. (**D**) Plotting ChIP peak signals using sequential ChIP-qPCR data from EGR1 binding site (S_1_-S_10_). Signal intensity was normalized to 1 in all the PRDM8-depleted bins. Projected peak height for control cells was calculated for every bin and smoothed spline fit was plotted. This plot showed significant decrease in normalized binding enrichment in PRDM8-depleted cells compared to the control cells. (**E**) IGV snapshot of the PRDM8 ChIP-seq performed using KO and control MCC-13 cells (two replicates) illustrating PRDM8 binding at EGR1 region. (**F**) EGR1 and PRDM8 expression levels in VN- vs. VP-MCC indicated higher levels of EGR1 and PRDM8 co-expression in VN-MCC compared to VP-MCC cells.

Top 5 TFs (YY1, CHAMP1, EGR1, TRIM22, and KDM1A) binding to PRDM8 were identified in eight analyzed samples, as demonstrated in the Figure 5A. Blue circles show TFs with high affinity to PRDM8. Aggregate number for each row is shown in the green horizontal bar next to each TF (Figure 5A). 

To experimentally validate binding of PRDM8 to the promoters of these TFs, we utilized PRDM8-depleted cells (MCC13_sgPRDM8) as well as PRDM8-intact cells (MCC13_CTRL) to investigate the TF binding alterations. PRDM8 chromatin immunoprecipitation (ChIP) on both cell lines followed by the analysis of the binding intensity at these TFs using quantitative PCR were performed (Figure 5B). To mark active promoters of the TFs, we utilized a previously known histone mark of active promoter, trimethylated H3K4 (H3K4me3 ChIP-seq in MCC, GSM1711864). Using the H3K4me3, as shown in Figure 5B (green box), we obtained the promoter binding site coordinates, split the sequence into ≈400 bp bins, and designed primer pairs specific to each bin of the binding sequence. Next, we assessed signal enrichment (indicating PRDM8 binding) in the chromatin-immunoprecipitated cells (ChIP-qPCR). Analysis of these data revealed that signal intensity was significantly decreased upon PRDM8 depletion only in EGR1 binding peak and no other TFs (Figure 5C,D and Figure S1D–H). To validate these findings, we performed PRDM8 ChIP-seq in MCC-13 cells and corresponding PRDM8-KO cells. We noticed absence of binding enrichment in PRDM8-KO cells at the EGR1 region (Figure 5E). In line with the finding that PRDM8 might work through EGR1, we interestingly noticed that EGR1 expression level was also correlated with its PRDM8 level and was higher in VN-MCC compared to VP-MCC (Figure 5F).

## 4. Discussion

This study reports the role of PRDM8 in virus-negative Merkel cell carcinoma. Here, we show that PRDM8 was upregulated in the VN-MCC compared to VP-MCC and its expression level was concordant with the global expression level of a major heterochromatin mark, trimethylated H3K9. Because H3K9me3 is considered as a histone mark that is widely associated with transcriptional repression, higher levels of this histone modification could have a global impact on the transcription machinery and can potentially repress gene transcription in a number of genes, which can ultimately lead to the unique characteristics of VN-MCC tumors that are known to be quite different from that of VP-MCC tumors [26,27]. It has previously shown that inactivation of tumor suppressors could be a mechanism of tumorigenesis in MCC. RB1 is a tumor suppressor that is frequently mutated in VN-MCC tumors [4,5,28]. The extent to which heterochromatin silencing of tumor suppressor genes contribute to tumorigenesis is not fully understood. Our findings in this study indicating PRDM8-induced increase in trimethylation of histone H3K9 might support this notion by playing a role in silencing tumor suppressor genes. We also demonstrated the role of PRDM8 in the tumor cell proliferation and clonogenicity in VN-MCC using the CRISPR-mediated loss-of-function assay and validated the dependency between the expression level of PRDM8 and H3K9me3. However, this has to be further examined by exploring tumor suppressor genes that are being impacted by such a mechanism.

Next, we identified miR-20a-5p to act as upstream regulator of PRDM8 that can target PRDM8 and modulate the transcriptional levels of this gene that will lead to the global repression of H3K9me3. We showed that in VN-MCC, expression level of this miRNA is suppressed, leading to subsequently high levels of PRDM8 and H3K9me3. Several studies have already shown the role of miRNAs including miR-375, miR-30a-3p, and miR-30a-5p induced by MCV T-antigens or MCV-miR-M1-5p encoded by MCV in VP-MCC [29,30,31]. MiR-9/9* is also shown to be expressed in MCC cells [32]. In this study, we identified miR-20a-5p as a downregulated miRNA in VN-MCC, which plays a role in transcriptional regulation of PRDM8. We demonstrated that miR-20a-5p, which can target PRDM8, was downregulated in VN-MCC vs. VP-MCC and ectopic overexpression of this miRNA could lead to decrease in PRDM8 expression level. Downregulation of PRDM8 that acts as a histone H3 methyltransferase will decrease trimethylation of H3K9. Interestingly, miR-9/9∗ and miR-124 are shown to trigger reconfiguration of chromatin accessibility [33], as deregulations in the H3K9me3 levels could also lead to chromatin accessibility alterations by changing heterochromatin content. Our findings in this study showed that silencing PRDM8 either through sgRNA-CRISPR knockout of the gene or miR-20a-5p overexpression could lead to global changes in heterochromatin pattern in VN-MCC cells, and that this could further reduce the tumorigenic capacity of the cells. Lastly, we demonstrated a possible PRDM8 function through binding to EGR1 (early growth response protein 1). We further functionally validated that depletion of VN-MCC cells from PRDM8 can lead to the loss of PRDM8–EGR1 binding, which could potentially decrease the MCC cells’ clonogenic capacity. 

Despite the findings described in this study, there are certain limitations in the studies involving MCC including ours that need to be overcome to be able to generalize any findings. There is still a very limited number of characterized cell lines (particularly VN-MCCs), in vitro models, or in vivo models that can be used in the experiments. However, there are a number of MCC cell lines, including those used in this study, which have been published previously and are available to the scientific community upon request. Because the cells of origin for MCC are still not clear, generating such cell line models and characterization of these models are still debatable. Moreover, due to the lower number of virus-negative MCC cases compared to VP-MCCs, recruiting more VN-MCC patients can also be challenging for such studies.

Taken together, our findings reveal a novel mechanism for the tumorigenesis of virus-negative MCC through the regulation of histone methyltransferase PRDM8, which is governed by the miR-20a-5p miRNA expression. Investigation of small RNA and miRNA-based strategies to regulated PRDM8 could be a potential path to the future therapies for VN-MCC.

## 5. Conclusions

This study demonstrates a histone methyltransferase role of PRDM8 in virus-negative MCC. We show higher expression levels of PRDM8 in VN-MCC cells as well as patient tumor samples. This finding prompted us to explore the role of this protein using loss-of-function experiments in *in vitro* MCC models, by which we identify the impact of this protein on proliferation and colony formation capacity in MCC cells. Further, we indicate the correlation of PRDM8 expression with H3K9me3 level. Moreover, our results show a miRNA regulatory mechanism that acts upstream of PRDM8 to modulate its expression, which is impaired in virus-negative MCCs.

## Supplementary Materials

The following are available online at https://www.mdpi.com/2072-6694/12/4/1057/s1, Figure S1: (**A**) PRDM8 protein expression assessed using Western blot on VP- and VN-MCC cell lines. (**B**) Western blotting a VN-MCC cell line (MCC13) showing PRDM8 knockdown influenced the H3K9me3 global expression level. Histone H3 was used as an internal control. (**C)** PRDM8 immunohistochemistry (IHC) intensity (score: 0–3) and quantity (score: 0–4) scores in VP- and VN-MCC patients. (**D**) Primers used in qPCR analysis of the ChIPed samples for the binding site region of EGR1. This site was split into 10 bins of ≈400bp (S_1_-S_10_). (**E**) IGV browser snippet from H3K4me3 ChIP-seq track of the MCC cells, showing promoter binding site around the TSS of YY1. Chromosome ideogram and coordinates are shown in the upper panel. Binding site was split in ≈400bp bins (S_1_–S_12_) to be analyzed using primers specific to each bin. (**F**) Control cells and PRDM8-depleted cells (MCC13_CTRL and MCC13_sgPRDM8) underwent PRDM8 chromatin immunoprecipitation followed by qPCR for bins in the binding site. Binding enrichment for PRDM8-depleted cells and control cells are shown in bins S_1_ to S_12_. MCC13_CTRL is in blue and MCC13_sgPRDM8 is in red. (**G**) IGV browser snippet from H3K4me3 ChIP-seq track of the MCC cells, showing promoter binding site around the TSS of CHAMP1. Chromosome ideogram and coordinates are shown in the upper panel. Binding site was split in ≈400bp bins (S_1_–S_7_) to be analyzed using primers specific to each bin. (**H**) Control cells and PRDM8-depleted cells (MCC13_CTRL and MCC13_sgPRDM8) underwent PRDM8 chromatin immunoprecipitation followed by qPCR for bins in the binding site. Binding enrichment for PRDM8-depleted cells and control cells are shown in bins S_1_ to S_7_. MCC13_CTRL is in blue and MCC13_sgPRDM8 is in red.

## Abbreviations

ChIP	Chromatin immunoprecipitation
DEG	Differentially expressed gene
ENCODE	Encyclopedia of DNA Elements
FDR	False discovery rate
FFPE	Formalin-fixed paraffin-embedded
GO	Gene Ontology
GSEA	Gene set enrichment analysis
IGV	Integrative Genomics Viewer
IHC	Immunohistochemistry
MCC	Merkel cell carcinoma
MCPyV	Merkel cell polyomavirus
MCV	Merkel cell polyomavirus
NES	Normalized enrichment score
TMA	Tissue microarray
VP-MCC	Virus-positive Merkel cell carcinoma
VN-MCC	Virus-negative Merkel cell carcinoma
