# Supplementary material for: Unique Role of Histone Methyltransferase PRDM8 in the Tumorigenesis of Virus-Negative Merkel Cell Carcinoma
Source: Cancers (Basel). 2020 Apr 24;12(4):1057. doi: 10.3390/cancers12041057 (PMC7226539; doi:10.3390/cancers12041057)
Supplement: Supplementary file 1 [file cancers-12-01057-s001.zip › cancers-767108-supplementary materials/cancers-767108-Figure S1.pdf]

Supplementary Materials

Unique role of histone methyltransferase PRDM8 in the tumorigenesis of virus-negative Merkel cell carcinoma

Elias Orouji, Wiebke K. Peitsch, Azadeh Orouji, Roland Houben, Jochen Utikal

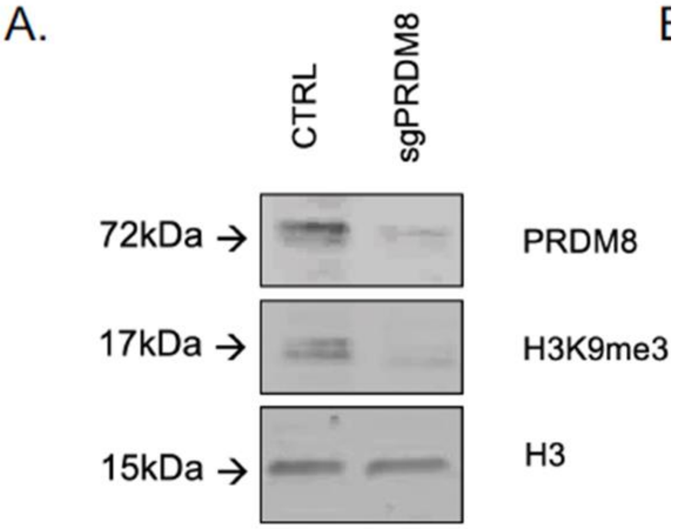

B.

		FWD primer (5'-3')	REV primer (5'-3')
EGR1	S1	CCTACCAGGGAGTGGCAAAA	TCACTCCCACTGTCTACCGA
	S2	CTTCCCGGAATCCCTGTTCC	GGATAACACTCCTCACCCCG
	S3	CTGCTCAGTTCGTGCTCACT	GCTTCCCTATGGGCTGTCTG
	S4	CAAGTGGCCGTGACTTCCTA	CGGGGTTCTATCGCTGTCAT
	S5	ACCTTATTTGGAGTGGCCCG	GCTTCGGGGAAGCCTAGAG
	S6	GGCCGGTCCTGCCATATTAG	GAACACTGAGAAGCGTGCAG
	S7	CCCGTTCGGATCCTTTCCTC	CCACTGCTTACCTGCGGTC
	S8	AGCCGGGTTTCCCTTTCATT	CCGCTCATCAAAACAAGCCC
	S9	CCGTATTTGCGTCAGCTGTT	GAGTGGTTTGGCTGGGGTAA
	S10	CCCCTCTTCAGCTTGGTCAG	CTGGAAGGGCTTCTGGTCTG

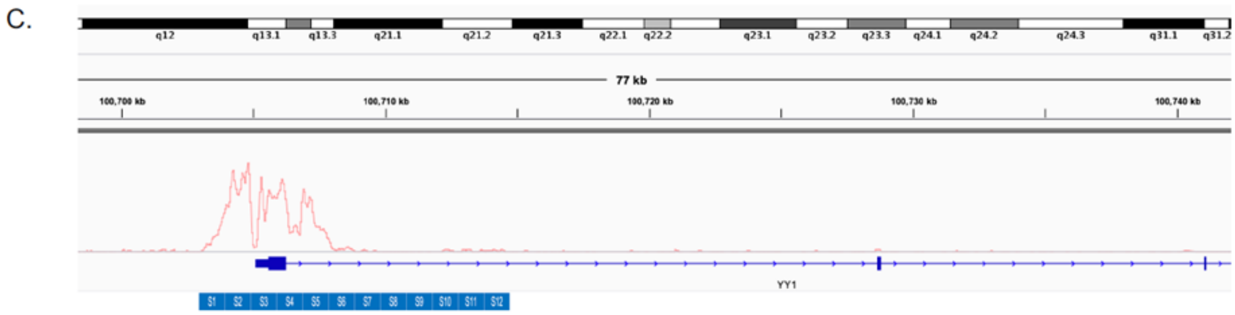

D.

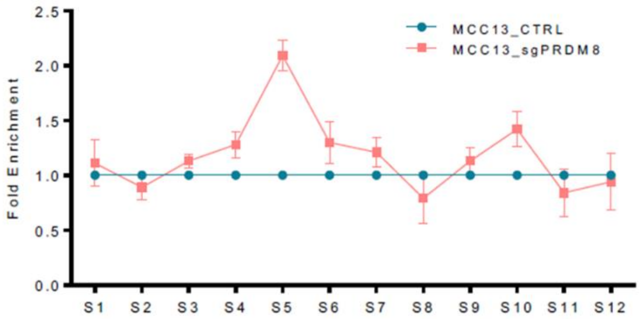

E.

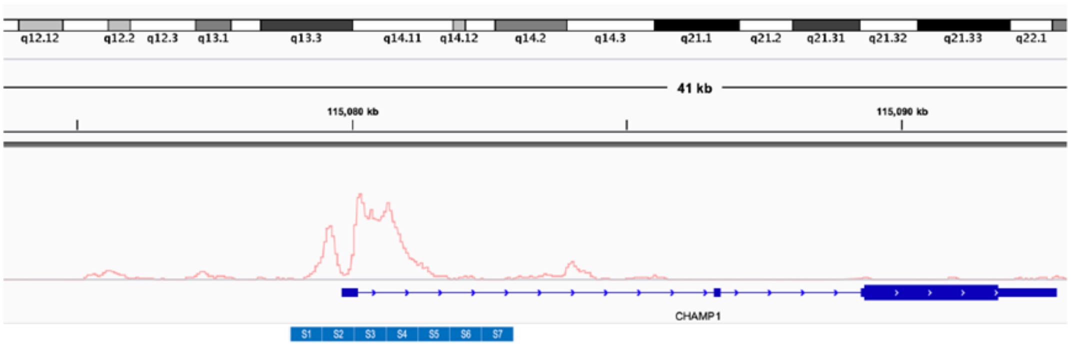

F.

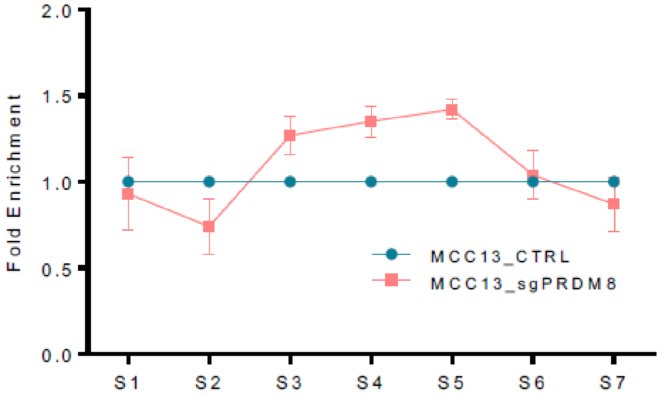

(A) Western blotting a VN-MCC cell line (MCC13) shows PRDM8 knockdown influences the H3K9me3 global expression level. Histone H3 is used an internal control. **(B)** Primers used in qPCR analysis of the ChIPed samples for the binding site region of EGR1. This site is split into 10 bins of ~400bp (S_1-S_{10}). **(C)** IGV browser snippet from H3K4me3 ChIP-seq track of the MCC cells, shows promoter binding site around the TSS of YY1. Chromosome ideogram and coordinates are shown in the upper panel. Binding site is split in ~400bp bins (S_1-S_{12}) to be analyzed using primers specific to each bin. **(D)** Control cells and PRDM8-depleted cells (MCC13_CTRL and MCC13_sgPRDM8) have undergone PRDM8 chromatin immunoprecipitation followed by qPCR for bins in the binding site. Binding enrichment for PRDM8-depleted cells and control cells are shown in bins S_1 to S_{12}. MCC13_CTRL is in blue and MCC13_sgPRDM8 is in red. **(E)** IGV browser snippet from H3K4me3 ChIP-seq track of the MCC cells, shows promoter binding site around the TSS of CHAMP1. Chromosome ideogram and coordinates are shown in the upper panel. Binding site is split in ~400bp bins (S_1-S_7) to be analyzed using primers specific to each bin. **(F)** Control cells and PRDM8-depleted cells (MCC13_CTRL and MCC13_sgPRDM8) have undergone PRDM8 chromatin immunoprecipitation followed by qPCR for bins in the binding site. Binding enrichment for PRDM8-depleted cells and control cells are shown in bins S_1 to S_7. MCC13_CTRL is in blue and MCC13_sgPRDM8 is in red.

© 2019 by the authors. Submitted for possible open access publication under the terms and conditions of the Creative Commons Attribution (CC BY) license (http://creativecommons.org/licenses/by/4.0/).
