# Supplementary figures and images for: Unique Role of Histone Methyltransferase PRDM8 in the Tumorigenesis of Virus-Negative Merkel Cell Carcinoma
Source: Cancers (Basel). 2020 Apr 24;12(4):1057. doi: 10.3390/cancers12041057 (PMC7226539; doi:10.3390/cancers12041057)

Western blot images:

Fig. 2E

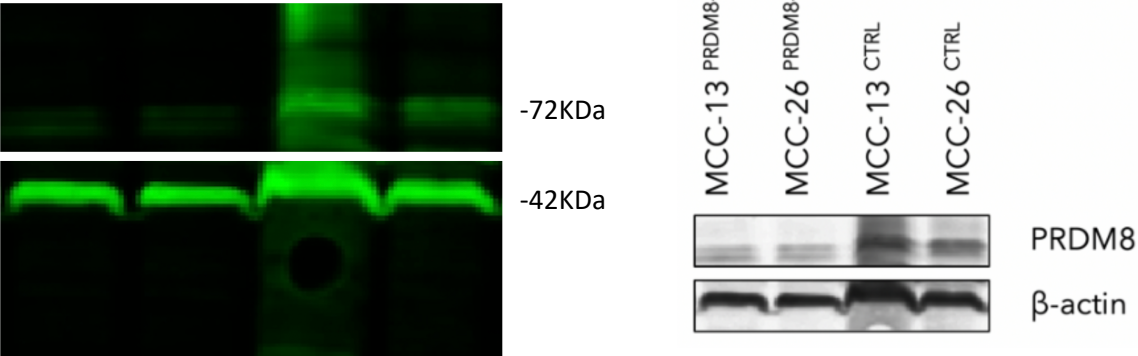

Fig. 4E

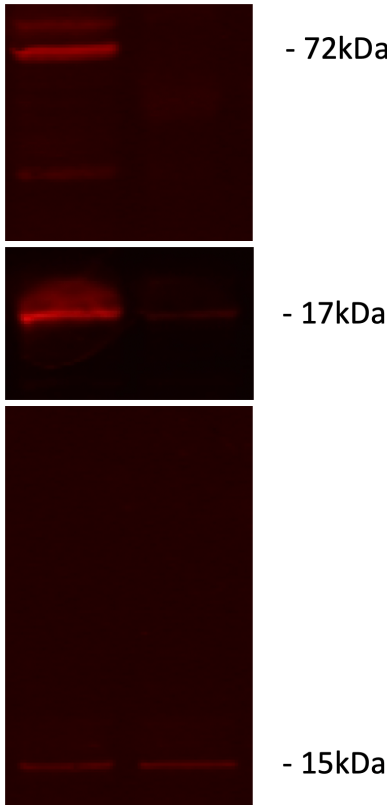

Suppl. Fig. 1A

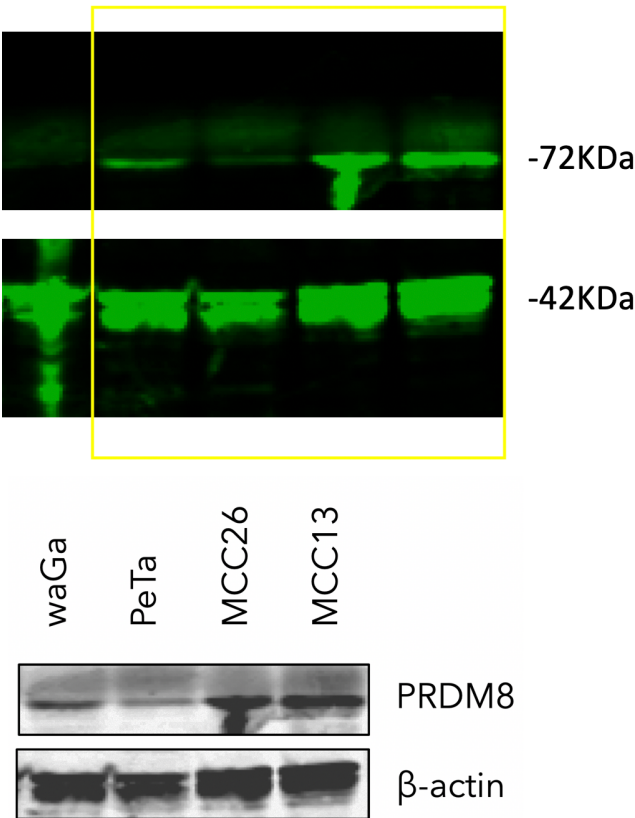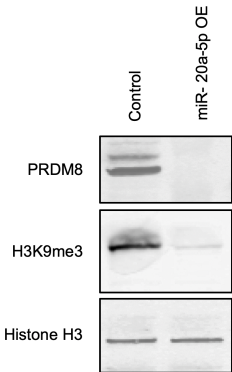

Suppl. Fig. 1B

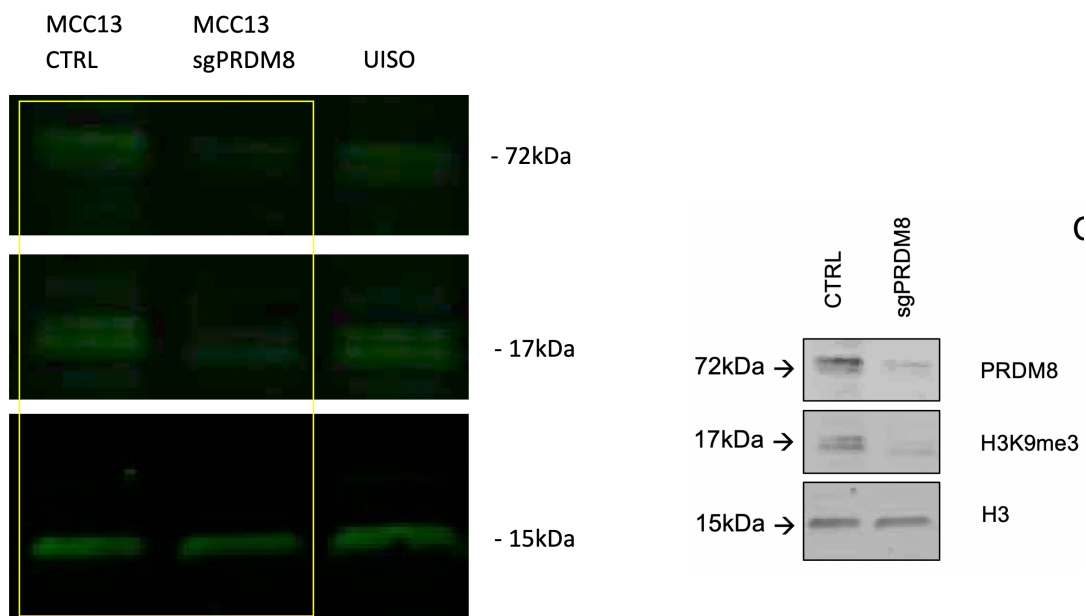

Densitometry for Suppl. Figures:

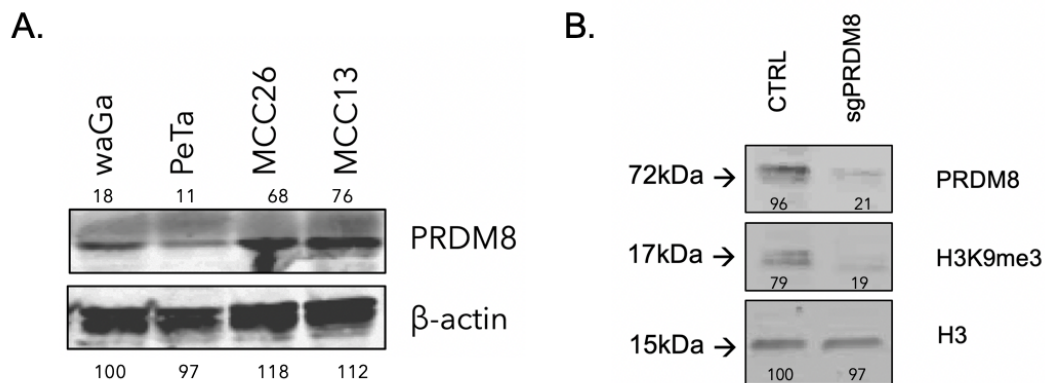

Supplement: Supplementary file 1 [file cancers-12-01057-s001.zip › cancers-767108-supplementary materials/cancers-767108-ucropped blot figures.pdf]
